# Curve of Spee modification in different vertical skeletal patterns after clear aligner therapy: a 3D set-up retrospective study

**DOI:** 10.1186/s40510-023-00503-1

**Published:** 2024-01-22

**Authors:** Domenico Ciavarella, Carlotta Fanelli, Carmela Suriano, Alessandra Campobasso, Mauro Lorusso, Donatella Ferrara, Marta Maci, Rosa Esposito, Michele Tepedino

**Affiliations:** 1 Department of Clinical and Experimental Medicine, Dental School of Foggia, University of Foggia, Via Rovelli, 50, 71122 Foggia, Italy; 2 Department of Biotechnological and Applied Clinical Sciences, University of L’Aquila, Via Lorenzo Natali, 67100 Coppito, L’Aquila, Italy

**Keywords:** Clear aligners, Curve of Spee depth, Divergence, Levelling

## Abstract

**Objective:**

The purpose of the present study was to evaluate: (1) changes in the curve of Spee (COS) after clear aligner therapy and (2) whether such changes correlated with the patient’s skeletal patterns.

**Materials and methods:**

Three-dimensional mandibular models of 106 patients (47 males and 59 females; mean age 22.3 SD ± 3.4 years) treated with clear aligners were retrospectively analysed. The perpendicular distance between the occlusal reference plane and the buccal cusp tip of each lateral tooth was measured. Five angular cephalometric measurements (PP-MP, PP-OP, OP-MP, AFH, and PFH) were performed and correlated with the T1–T0 difference in linear distances. The patients were divided into three groups according to facial divergence. Variance analysis with Tukey post hoc correction was performed to evaluate the differences among groups.

**Results:**

The one-way ANOVA test showed a statistically significant difference for all analysed variables, except for the change in the distance of the second molar from the occlusal reference plane. Tukey’s HSD test showed the following differences: In normodivergents, the T1–T0 difference in the distance of the first molar from the occlusal plane was 1 mm greater than that observed in the hyperdivergent group (*p* < 0.05); in the normodivergent group, the T1–T0 difference in the distance of the second premolar from the occlusal plane was 1.23 mm greater than that observed in the hyperdivergent group (*p* < 0.05), while in the hypodivergent group, it was 1.08 mm greater than in the hyperdivergent group (*p* < 0.05); finally, in normodivergents, the T1–T0 difference in the distance of the first premolar from the occlusal plane was 0.97 mm greater than that observed in the hyperdivergent group (*p* < 0.05).

**Conclusion:**

Treatment with aligners did not lead to a clinically significant change in COS depth. However, when dividing the sample into groups based on craniofacial divergence, COS depth change differed significantly between the three groups.

## Introduction

The curve of Spee (COS) was defined as the anatomic curve established by the occlusal alignment of teeth, beginning with the cusp of the canine and following the buccal cusp tips of the premolar and molar teeth continuing through the anterior border of the mandibular ramus and ending at the anterior aspect of the mandibular condyle) [1]*.* COS levelling is a fundamental objective during orthodontic treatment, following Andrews’ six keys for achieving optimal occlusion [2].

In fixed orthodontic treatment, the levelling of the COS, achieved through the placement of brackets and archwires, enables modification of the vertical position of the posterior teeth, consequently modifying the anterior overbite [3]. The COS shape differs between deep bite and open bite patients [4]. An excessive COS is associated with deep bite malocclusions, while a reverse curve is typical in open bite cases. In order to establish a stable occlusion with appropriate excursive movements, the curve needs to exhibit a relatively mild configuration. The levelling of the COS may involve the intrusion of mandibular anterior teeth, the extrusion of mandibular posterior teeth, or a combination of both movements [5]. The levelling effect achieved in orthodontic treatment to correct both deep bite and open bite conditions, using various methods of tooth extrusion or intrusion, has been evaluated in numerous studies [6–8]. According to several authors, the COS should be corrected by employing molar extrusion. This approach is preferred due to the high potential for relapse associated with intruded anterior teeth [9, 10].

Requests for aesthetic treatment in adults have led to orthodontists using clear aligner treatment (CAT) [11]. The predictability of mandibular COS levelling using aligner appliances has been explored by only a few authors, and the conclusions drawn from these studies remain controversial [12, 13]. Some authors have reported the effectiveness of levelling through incisor intrusion with proclination control [12], while others have observed significant overestimation in mandibular COS levelling when checking the virtual set-up [13].

The bite-block effect provided by CAT and the thickness of the aligner plastic, in combination with occlusal forces, could determine the intrusion of the posterior teeth, making it effective in resolving open bite malocclusions [14].

CAT is frequently utilized to treat mild crowding in patients without severe open or deep bite and with various skeletal patterns [15]. It is questionable whether the bite-block effect can induce a change in the depth of COS in these patients.

Extended use of CAT has been associated with potential alterations in the orientation of the occlusal plane [16] and subsequent improvements in muscular function due to enhanced occlusal contact [17].

Despite the increasing use of clear aligners, there is a lack of studies examining the impact of these appliances on COS when addressing moderate crowding without actively prescribing dental movements that would change the COS.

The aim of the present study was to evaluate the changes in COS during CAT in patients with upper and lower crowding, within a treatment plan that did not involve the extrusion or intrusion of posterior and anterior teeth. As a secondary outcome, the change in COS in different skeletal divergence patterns was compared. The null hypothesis was that no difference exists between pre- and post-treatment COS depth, and that no difference exists in COS between the different facial divergence patterns.

## Material and methods

This study was reported following the Strengthening the Reporting of Observational Studies in Epidemiology (STROBE) guidelines for observational studies [18].

All the procedures of this research protocol adhered to the Declaration of Helsinki and were approved by the Ethics Committee of the University of Foggia. The records were retrieved retrospectively and analysed anonymously, and patients signed a written informed consent. The inclusion and exclusion criteria are listed in Table 1.

**Table 1:** Inclusion and exclusion criteria

Inclusion criteria	Exclusion criteria
Non growing patients (skeletal age CS6 according to the cervical vertebral maturation method)	Use of auxiliaries in combination with clear aligners
Non extraction treatment	Periodontal disease
Treatment with a clear aligner system with a thickness of 0.75 mm	Implants or root canal therapies before and during the orthodontic treatment
Sequence of 15 aligners or more	Temporomandibular disorders
Moderate crowding (4–6 mm) according to the Little index	Skeletal malformations and destructive caries

Crowding was assessed using Little’s index, which measures the distance between the anatomical contact points of the anterior teeth [19].

A power analysis (G*Power 3.1.9.2, Franz Faul, Universitat Kiel, Germany) revealed that to detect a large effect size of 0.4 [20] with a one-way ANOVA test, an *α* error probability of 0.05 and a power (1 − *β* error probability) of 0.95, 84 subjects would be needed.

This study involved 106 Caucasian patients (47 males and 59 females; mean age 22.3 SD ± 3.4 years) with Class I malocclusion who were treated with clear aligners. All aligners used in the present study were made by the same provider and used the same material. The treatment focused on avoiding both molar and incisor extrusion or intrusion, as well as preventing incisor proclination, to address the malocclusion. Moderate crowding was resolved through interproximal enamel reduction (IPR).

The sample, for descriptive purposes, was divided into three groups according to the values of the angle SN-MP:

- *Group 1* SN-MP > 35.5° (34 hyperdivergent subjects);
- *Group 2* 30.5 ≤ SN-MP ≤ 35.5° (36 normodivergent subjects);
- *Group 0* SN-MP < 30.5° (36 hypodivergent subjects)

These values represent one standard deviation (SD) from the average SN-MP angle reported by the Italian Board of Orthodontics (IBO) and European Board of Orthodontics (EBO) [21]. The groups were retrospectively enrolled from patients treated at the Department of Orthodontics, University of Foggia, in chronological order from April 2017 to November 2019. Pre-treatment (T0) and post-treatment (T1) records included a digital scan of the dental arches and lateral cephalograms.

**Fig. 1:**
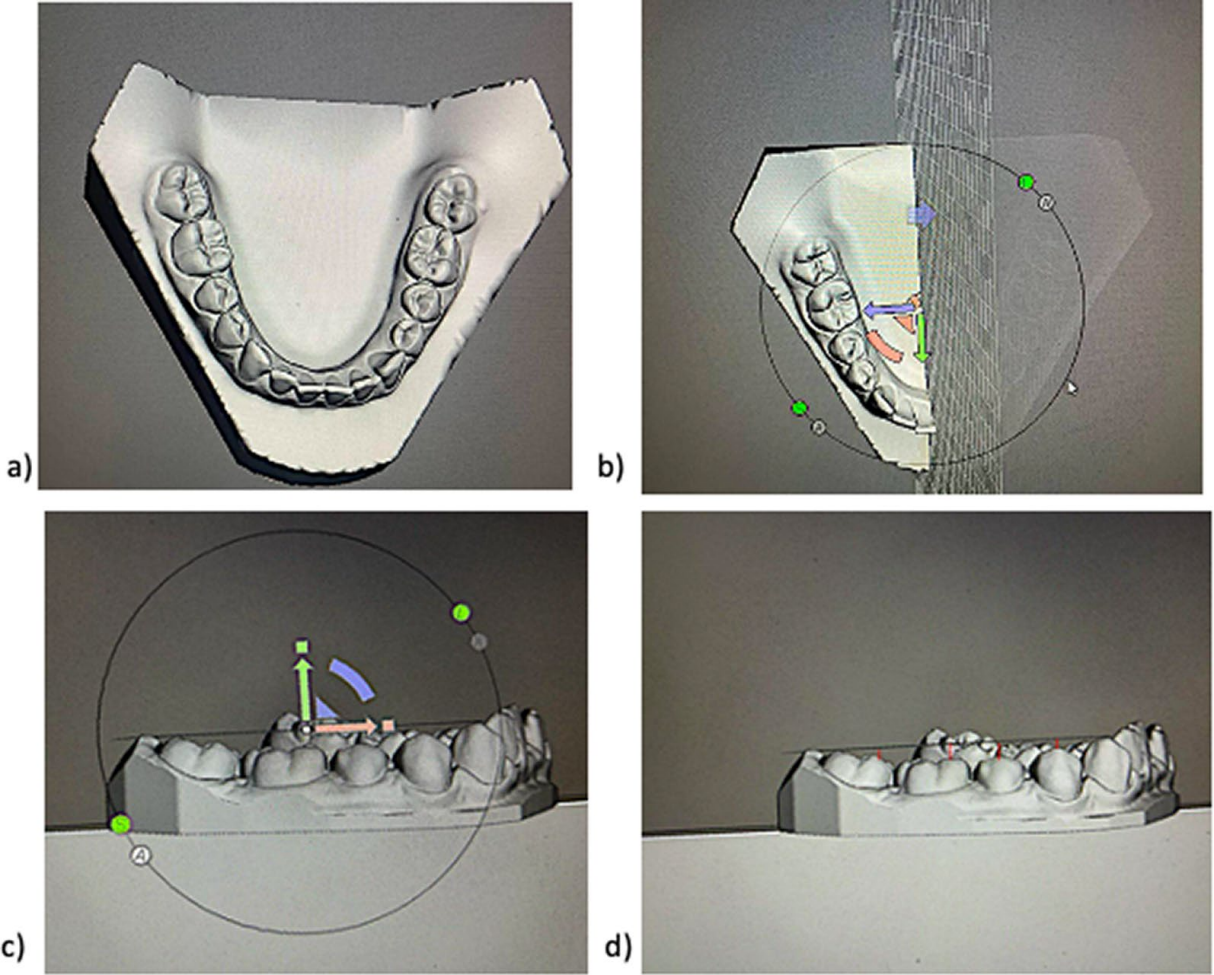
Procedure for COS depth measurements on digital models. **a** Occlusal view of mandibular stl model, **b** occlusal view and selection of right side of jaw, **c** sagittal view of model and occlusal reference passing through the cusp of canine and buccal distal cusp of mandibular second molar and **d** measurement of the distance from the occlusal reference plane to each tooth in order to determine the depth of the COS

A direct scan of the maxillary and mandibular arches was performed before and after treatment using an intraoral scanner (TRIOS; 3Shape, Copenhagen, Denmark), following the protocol recommended by the manufacturer. The standard triangle language (STL) files were imported into dental CAD software (Meshmixer, Autodesk Inc.) to generate virtual models. An occlusal plane reference was established by drawing a line passing through the distobuccal cusp of the mandibular second molar on the right side and the cusp of canine anteriorly [22–24]. Using Ortho Analyzer (3Shape), the perpendicular distance between this plane and the buccal cusp tip of each lateral tooth was measured specifically on the right side of the curve (13). These linear values were measured both before (T0) and after (T1) CAT to obtain the depth comparison Δ (T1–T0) for the second molar (Δ7MB), first molar (Δ6MB), second premolar (Δ5B), and first premolar (Δ4B). The dental measurements were performed for each digital scan of the mandibular arches and are described in Fig. 1.

### Cephalometric analysis

Lateral head cephalogram and digital scans were performed for each patient pre-treatment, and the SN-MP angle was measured.

All the lateral radiographs were taken by the same technician on the same machine (Gendex GXDP-700) in the same radiology department. To minimize measurement errors, a trained orthodontist examiner performed the cephalometric analyses and dental measurements. The following cephalometric variables, described in Table 2, were analysed: ANB, PP-MP, PP-OP, OP-MP, AFH, and PFH.

**Table 2:** List and definition of all the measurements used in the present study

Measurement	Definition
Angular (°)	
PP-MP	The angle between the palatine plane (PP), passing through Ans and Pns, and mandibular plane (MP)
PP-OP	The angle between the palatine plane (PP), passing through Ans and Pns, and occlusal plane
OP-MP	The angle between the occlusal plane (OP), passing through the incisal edge of U1 to the midpoint of the U6 on the occlusal, and mandibular plane (MP)
AFH	The anterior facial height (Na-Me) expressed in percentage
PFH	The posterior facial height(S-Go)
Linear (mm)	
Δ7MB	Post-treatment difference in the linear distance from the mesio-buccal cusp of the second mandibular molar to the reference plane
Δ6MB	Post-treatment difference in the linear distance from the mesio-buccal cusp of the first mandibular molar to the reference plane
Δ5B	Difference after treatment linear distance between buccal cusp of second premolar and reference plane
Δ4B	Difference after treatment linear distance between buccal cusp of the first premolar and reference plane

### Statistical analysis

To reduce random errors, cephalometric and dental measurements were taken twice. The random error of each measurement was calculated using Dahlberg’s formula (*S* = ∑ *d*^2^/2*N*), where *d* is the difference between the first and second measurements and *N* is the number of radiographs evaluated [25, 26]. The random error of cephalometric measurements ranged between 0.12 and 0.31 mm for linear measurements and between 0.38° and 0.76° for angular measurements. The random error of dental measurements ranged between 0.11 and 0.23 mm.

Data were analysed using GraphPad Prism software 6.0 (GraphPad Prism Software, San Diego, CA, USA). The Shapiro–Wilk normality test was conducted to evaluate data distribution (Table 3). Descriptive statistics were also performed (Tables 4, 5). To assess differences between the three groups, a one-way ANOVA test was conducted on the T1−T0 difference of each variable, followed by Tukey’s post hoc test (Tables 6, 7) for variables with homogeneous variances. For the non-homogeneous variables (Δ7MB, Δ6MB, and Δ5B), a Welch-ANOVA test was performed followed by the Games–Howell post hoc test. Statistical significance was set as *p* < 0.05.

**Table 3:** Results of the Shapiro–Wilk normality test for all variables of the whole sample and by group

Variable	Cohort	Statistics	*df*	*p*
△7 MB	Normodivergent (*n* = 36)	0.962	34	0.278
	Hyperdivergent (*n* = 34)	0.959	28	0.334
	Hypodivergent (*n* = 34)	0.834	34	0.000
	Total sample (n = 106)			
△6 MB	Normodivergent (*n* = 36)	0.947	34	0.097
	Hyperdivergent (*n* = 34)	0.967	28	0.500
	Hypodivergent (*n* = 34)	0.896	34	0.004
	Total sample (*n* = 106)			
△5B	Normodivergent (*n* = 36)	0.946	34	0.096
	Hyperdivergent (*n* = 34)	0.945	28	0.145
	Hypodivergent (*n* = 34)	0.956	34	0.181
	Total sample (*n* = 106)			
△4B	Normodivergent (*n* = 36)	0.935	34	0.044
	Hyperdivergent (*n* = 34)	0.925	28	0.047
	Hypodivergent (*n* = 34)	0.962	34	0.276
	Total sample (*n* = 106)

**Table 4:** Descriptive statistic of COS modification among the groups

Cohort	Mean dept T0 (mm)	Mean dept T1 (mm)	Mean dept ΔT1 − T0 (mm)
Normodivergents (*n* = 36)	1.64	2.09	0.45
Hyperdivergents (*n* = 34)	2.02	1.48	−0.54
Hypodivergents (*n* = 34)	2.11	2.13	0.02
Total sample (*n* = 106)	1.92	1.90	−0.01

**Table 5:** Descriptive statistics of each group at time T0 and time T1

	7MV T0		6MV T0		5M T0		4M T0		7MV T1		6MV T1		5M T1		4M T1	
	Mean	SD	Mean	SD	Mean	SD	Mean	SD	Mean	SD	Mean	SD	Mean	SD	Mean	SD
Normodivergent group	− 0.08	1.73	1.64	1.36	1.05	1.74	0.35	1.21	0.39	1.65	2	1.35	1.28	1.2	0.66	1.46
Hyperdivergent group	0.16	1.52	2.02	1.52	1.07	1.51	0.40	1.69	0.24	1.94	1.59	1.48	0.47	1.31	− 0.4	1.71
Hypodivergent group	0.48	1.37	2.11	1.62	1.48	1.06	0.93	1.16	0.56	1.76	2.16	1.68	1.67	1.42	0.85	1.41

**Table 6:** One-way ANOVA test for COS depth measurements between the three groups

	Sum of squares	*df*	Mean square	*F*	Sig.
∆7MB					
Between groups	1.32	2	0.66	0.19	0.827
Within groups	337.42	97	3.48		
Total	339.75	99			
∆6MB					
Between groups	16.57	2	8.28	5.16	0.007**
Within groups	155.55	97	1.60		
Total	172.13	99			
∆5MB					
Between groups	29.69	2	14.84	5.64	0.005**
Within groups	254.96	97	2.62		
Total	284.65	99			
∆4B					
Between groups	16.13	2	8.06	3.16	0.04*
Within groups	252.06	99	2.54		
Total	268.19	101			

*p < 0.05; **p < 0.01

**Table 7:** Tukey’s post hoc test and Games–Howell post hoc test

Dependent variable	(I) Group	(J) Group	Mean difference (I − J)	Std error	*p*	95% Confidence interval	
						Lower bound	Upper bound
∆7MB	Normodivergent	Hyperdivergent	0.20	0.49	0.909	− 0.98	1.40
	Normodivergent	Hypodivergent	0.26	0.42	0.804	− 0.74	1.28
	Hypodivergent	Hyperdivergent	0.05	0.45	0.991	− 1.15	1.03
∆6MB	Normodivergent	Hyperdivergent	1*	0.33	0.010	0.20	1.79
	Normodivergent	Hypodivergent	0.43	0.31	0.373	− 0.33	1.2
	Hypodivergent	Hyperdivergent	0.56	0.27	0.101	1.22	1.22
∆5MB	Normodivergent	Hyperdivergent	1.23*	0.45	0.023	0.13	2.32
	Normodivergent	Hypodivergent	0.14	0.38	0.922	− 0.77	1.06
	Hypodivergent	Hyperdivergent	1.08*	0.34	0.008	0.24	1.93
∆4B	Normodivergent	Hyperdivergent	0.97*	0.38	0.036	0.05	1.89
	Normodivergent	Hypodivergent	0.49	0.38	0.410	− 0.42	1.41
	Hypodivergent	Hyperdivergent	0.47	0.38	0.434	− 0.44	1.39

*p < 0.05

## Results

Table 4 shows the changes in COS in the whole sample and in the three groups:

- The whole sample showed a non-relevant modification of the COS (− 0.01 mm).
- Hyperdivergent patients presented a slight flattening of the COS (0.5 mm).
- Hypodivergent patients presented a non-relevant alteration of the COS (0.02 mm).
- Normodivergent patients showed a negligible modification of the COS with an increase in its depth (0.45 mm).

When comparing the COS depth change between the three groups, one-way ANOVA (Table 6) and the WELCH-ANOVA showed a statistically significant difference for all analysed variables except for ∆7MV. Post hoc tests (Table 7) showed statistically significant differences as follows:

- ∆6 MB was 1 mm greater in normodivergent group than in hyperdivergent group;
- ∆5 MB was 1.23 mm greater in normodivergent group than in hyperdivergent group and was 1.08 mm greater in hypodivergent group than hyperdivergent group;
- △4 V was 0.97 mm greater in normodivergent group than in hyperdivergent group.

## Discussion

The present study investigated alterations in the COS among patients with upper and lower crowding and different vertical patterns who underwent CAT. The treatment approach involved an alignment set-up, excluding any additional corrections such as COS levelling. The implementation of new digital technologies in orthodontics has led to significant contributions across various aspects of clinical practice and research. These advancements encompass improved diagnosis and treatment planning, as well as enhanced outcome evaluation [27]. Dedicated 3D software has facilitated data acquisition and processing, allowing more accurate measurements to be obtained in a shorter time.

Measurements were performed without considering the patient’s sex. This approach was guided by published findings that have established the independence of the deepest point of the COS according to both sex and side [28, 29].

The sample considered in the present study did not require COS correction, as the deepest midpoint of the COS measured 2.2 mm before treatment. This observation is consistent with other authors who also considered a maximum of 2 mm as a normal COS measurement [22, 30, 31]. Moreover, in the present study, the deepest COS was found in the hypodivergent group. This finding aligns with the existing literature, which has demonstrated the influence of the ratio between posterior and anterior facial height, as well as divergence, on the COS [3].

The management of the COS is crucial for orthodontists as it plays a critical role not only in the diagnostic process [32, 33], but also in treatment planning. It is essential for achieving the correction and stabilization of a proper occlusion [34, 35]. Limited data are available regarding the efficacy of clear aligners in levelling the COS. However, it should be noted that clear aligners are generally less effective at achieving molar extrusion [12]. Clear aligners are frequently utilized to address moderate crowding without incorporating explicit intrusion or extrusion planning information in the modification of the COS. Although clear aligners are increasingly utilized in this type of treatment, limited research has been conducted to investigate aspects related to potential unplanned effects of this appliance on the COS [36].

According to the results of the present study, CAT did not cause any clinically or statistically significant alteration of the COS during treatment. However, when the sample was divided into groups based on craniofacial divergence, differences in COS stability after treatment could be detected.

In normodivergent patients, the distance between the reference occlusal plane and the vestibular cusps of the first premolar (Δ4B = 0.974 mm, *p* < 0.05), second premolar (Δ5B = 1.234 mm, *p* < 0.05), and first molar (Δ6MB = 1.00085 mm, *p* < 0.05) increased compared to hyperdivergent patients after CAT. The greater distance of the premolar and first molar cusps from the occlusal plane indicates an intrusion of these teeth in the normodivergent group. Additionally, a significant difference was observed between the hypodivergent and hyperdivergent groups regarding the distance of the second premolar to the occlusal plane. This data suggested intrusion of the second premolar in hypodivergent patients compared to the hyperdivergent groups after CAT.

No statistically significant differences were observed between normodivergent and hypodivergent groups, or between hypodivergent and hyperdivergent groups, in relation to the molars and first premolar. These data suggest different effects on the COS in the three groups when using aligners to address simple crowding. Specifically, in normodivergent and hypodivergent patients, the COS appears deeper in the initial models, whereas in hyperdivergent patients, the COS does not change significantly. Clear aligner treatment may be the preferred option for hyperdivergent patients, as it allows for easier control of the vertical dimension. However, it should be noted that CAT may result in a worsening of the initial vertical dimension in normodivergent and hypodivergent patients. The observed effects on the COS may be attributed to the occlusal contacts changing and the response of masticatory muscles induced by CAT. These factors result in occlusal stimulation during activities such as swallowing, speaking, and mandibular movements. Some authors have reported that CAT can induce variations in occlusal contact. Sultana et al. [37] suggested that using CAT only during the night can lead to a functional accommodation of occlusion, increasing the number of occlusal contacts. Dincer and Aslan [38] evaluated the occlusal contacts in patients wearing thermoplastic retainers at night for 9 months, and then again after 2.5 years. They reported a significant increase in the total number of occlusal contacts after 2.5 years of retention. According to Tepedino et al. [17], orthodontic treatment with clear aligners resulted in a sagittal shift in the centre of force (COF), moving it posteriorly when aligners were worn. Moreover, Marcellino et al. [39] observed differences in occlusal contact among different vertical patterns of growth after CAT. They reported that in hypodivergent and normodivergent patients, the anterior occlusal contacts were higher than planned, compared to hyperdivergent patients. Furthermore, Charalampaski et al. [40] reported that the presence of premature contacts in the anterior area, combined with the thickness of clear aligners, is one of the factors that can result in the loss of posterior contacts during CAT, thus promoting a bite-block effect.

It is well known that hypodivergent and normodivergent patients have an anteriorly placed occlusal barycentre compared to hyperdivergent patients. Moreover, the literature suggests that muscular soreness may occur after wearing clear aligners in the short term [41]. It could be hypothesized that the modification of occlusal stress induced by aligners may lead to immediate muscular changes and alterations in tooth position, resulting in COS changes with clear aligner treatment. The intrusion of molar and premolars in hypodivergent and normodivergent patients could be attributed to the more anterior placement of the occlusal barycentre in conjunction with greater muscle strength compared to hyperdivergents. Because of the presence of a thickness all along the occlusal surface from anterior to posterior teeth, it is reasonable to expect an increase in posterior contact with a decrease in anterior ones. A posterior shift of aligner region stressed leads to intrusion of posterior teeth.

Regarding second molars, no significant changes were observed after therapy. In the groups of analysed patients, the occlusal barycentre was positioned at different levels, and as a result, the muscle biotype had different effects on tooth movements. Second molars underwent some changes, with a slight intrusion in all biotype, but these movements were not statistically significant. (Δ7MB in hypodivergent 0.08 mm, Δ7MB in hyperdivergent 0.08 mm, and Δ7MB in normodivergent 0.47 mm). This could be due to the fact that the range of action of the aligners was concentrated in a smaller area, mainly involving the lateral teeth from the first premolar to the first molar.

The results of the present work suggest that CAT could not be the best choice for hypodivergent patients where CAT could result in a reduction in the initial vertical dimension.

### Limitation of the study

The retrospective nature of patient recruitment may have introduced inherent bias; however, efforts were made to minimize selection bias by strictly adhering to a chronological criterion. Additionally, the small sample size limits the generalizability of the findings to a larger population. Another limitation relates to the technique used for digital measurements, as it requires operators with expertise in digital programmes.

Additional studies are necessary to gain a comprehensive understanding of the effects of CAT on the changes in the curve of Spee.

## Conclusion

The present study evaluated the COS after CAT in patients with different craniofacial divergence patterns of growth. Aligner treatment did not result in a clinically significant modification of the COS after a mean treatment duration of 13 months. However, after dividing the sample into groups based on craniofacial divergence, the following results were observed:

- Hyperdivergent patients showed minimal changes in the COS, which were in line with the treatment plan and considered favourable;
- Normodivergent patients showed greater intrusion of the first and second premolar and first molar compared to hyperdivergent patients;
- Hypodivergent patients showed greater intrusion of the second premolar compared to hyperdivergent patients.

## Data Availability

The datasets used and/or analysed during the current study are available from the corresponding author on reasonable request.
