# Correction to: Curve of Spee modification in different vertical skeletal patterns after clear aligner therapy: a 3D set-up retrospective study

**DOI:** 10.1186/s40510-024-00528-0

**Published:** 2024-06-07

**Authors:** Domenico Ciavarella, Carlotta Fanelli, Carmela Suriano, Alessandra Campobasso, Mauro Lorusso, Donatella Ferrara, Marta Maci, Rosa Esposito, Michele Tepedino

**Affiliations:** 1 Department of Clinical and Experimental Medicine, Dental School of Foggia, University of Foggia, Via Rovelli, 50, Foggia, 71122 Italy; 2 Department of Biotechnological and Applied Clinical Sciences, University of L’Aquila, Via Lorenzo Natali, Coppito, L’Aquila, 67100 Italy

Correction to: *Prog Orthod.***25**, 5 (2024)

10.1186/s40510-023-00503-1.

Following publication of the original article [1], the authors identified an error in the author names of the author group as the given name and family name were erroneously transposed.

The incorrect author names are: Ciavarella Domenico, Fanelli Carlotta, Suriano Carmela1, Campobasso Alessandra, Lorusso Mauro, Ferrara Donatella, Maci Marta, Esposito Rosa and Tepedino Michele

The correct author names are: Domenico Ciavarella, Carlotta Fanelli, Carmela Suriano, Alessandra Campobasso, Mauro Lorusso, Donatella Ferrara, Marta Maci, Rosa Esposito and Michele Tepedino

The author group has been updated above and the original article [1] has been corrected.
